# Antimicrobial Susceptibility of Genital Ureaplasma spp. and *Mycoplasma hominis*

**DOI:** 10.3390/jcm15208129

**Published:** 2026-10-20

**Authors:** Mirkan Bulğak, Pınar Tuğçe Özer

**Affiliations:** 1 Department of Infectious Diseases and Clinical Microbiology, Faculty of Medicine, İzmir University of Economics, Medical Point Hospital, İzmir 35330, Türkiye; 2 Department of Obstetrics and Gynecology, İzmir University of Economics Medical Point Hospital, İzmir 35575, Türkiye; pintugbar@gmail.com

**Keywords:** Ureaplasma spp., *Mycoplasma hominis*, antimicrobial resistance, antimicrobial susceptibility, phenotypic surveillance, urogenital specimens

## Abstract

**Background/Objectives:** Ureaplasma spp. and *Mycoplasma hominis* may be detected as colonizers or in association with urogenital disease, while antimicrobial resistance and methodological variability complicate interpretation of susceptibility data. This study aimed to characterize local in vitro phenotypic antimicrobial susceptibility patterns of Ureaplasma spp. and *M. hominis* and to explore organism- and sex-associated differences. **Methods:** In this retrospective microbiological surveillance study, 242 patients were tested between April 2023 and April 2025 using the Mycoplasma IES commercial culture-based system. Specimens with insufficient growth or mixed Ureaplasma spp./*M. hominis* growth were excluded, leaving 176 single-organism-positive patients with valid susceptibility results. Eleven antimicrobial agents were evaluated using the assay-defined categorical interpretation. **Results:** Ureaplasma spp. were detected in 145 patients (82.4%) and *M. hominis* in 31 (17.6%). Phenotypic resistance was highest for clindamycin (99.4%), ciprofloxacin (91.5%), and ofloxacin (64.2%), whereas susceptibility was high for josamycin (99.4%), minocycline (98.9%), pristinamycin (98.9%), and tetracycline (84.1%). *M. hominis* showed substantially greater resistance than Ureaplasma spp. to clarithromycin, erythromycin, roxithromycin, and levofloxacin (all *p* < 0.001). Unadjusted sex differences in macrolide resistance were considered exploratory because *M. hominis* occurred almost exclusively among women. **Conclusions:** The findings provide local assay-derived phenotypic surveillance data and demonstrate marked organism- and antimicrobial-specific variation. Because the commercial assay does not distinguish U. urealyticum from U. parvum and reference MIC confirmation was not performed, the results should be interpreted within these methodological limitations and should not be used alone to infer clinical treatment efficacy.

## 1. Introduction

*Mycoplasma hominis* and Ureaplasma species are among the smallest self-replicating bacteria and are characterized by the absence of a cell wall. This structural feature confers intrinsic resistance to antimicrobial agents targeting cell-wall synthesis, including β-lactams and glycopeptides, thereby restricting therapeutic options to antimicrobial classes acting on other cellular targets [1,2]. *M. hominis*, Ureaplasma urealyticum, and Ureaplasma parvum may colonize the lower urogenital tract, particularly in sexually active individuals, without necessarily causing clinical disease. Under certain circumstances, however, these organisms have been associated with urethritis, reproductive-tract disorders, adverse pregnancy outcomes, and neonatal complications [3,4,5,6].

Importantly, detection of these organisms does not necessarily indicate clinically significant infection. Asymptomatic carriage of *M. hominis* and Ureaplasma spp. is common, and European expert guidance has emphasized that indiscriminate testing and treatment may result in unnecessary antimicrobial exposure and contribute to antimicrobial resistance [7]. Therefore, microbiological findings should be interpreted in the context of clinical presentation, indication for testing, organism burden, and the presence of other urogenital pathogens.

The absence of a cell wall limits potentially active antimicrobial classes primarily to tetracyclines, macrolides, fluoroquinolones, lincosamides, and selected other protein-synthesis inhibitors. Susceptibility differs substantially between genital mycoplasma species. In particular, *M. hominis* demonstrates intrinsic resistance to several 14- and 15-membered macrolides, whereas Ureaplasma spp. generally demonstrate a different susceptibility profile [8,9,10,11,12,13]. Acquired antimicrobial resistance may additionally arise through alterations involving ribosomal targets, tetracycline resistance determinants, and mutations within the quinolone resistance-determining regions of DNA gyrase and topoisomerase IV [12,14].

Antimicrobial resistance among urogenital Mycoplasma and Ureaplasma isolates has become an increasing concern worldwide. Surveillance studies and recent systematic reviews demonstrate substantial variation in resistance rates across geographic regions, study populations, antimicrobial agents, and susceptibility-testing methodologies [8,9,10,11,12,14,15,16]. This geographic and methodological heterogeneity limits the applicability of resistance estimates obtained in one population to another and highlights the importance of contemporary local surveillance.

Interpretation of susceptibility data is further complicated by differences among laboratory methods. Standardized reference methods, including broth microdilution and agar dilution approaches described by the Clinical and Laboratory Standards Institute (CLSI), provide MIC-based susceptibility assessment, whereas commercial culture-based systems may generate phenotypic categorical results using predefined antimicrobial concentrations [17]. Consequently, susceptibility rates generated by different methodologies may not be directly interchangeable.

Despite increasing international surveillance, contemporary local data describing antimicrobial susceptibility among genital mycoplasmas remain limited in many settings. Furthermore, organism-specific susceptibility patterns may be obscured when *M. hominis* and Ureaplasma spp. are analyzed together. The primary objective of the present study was therefore to characterize the in vitro phenotypic antimicrobial susceptibility patterns of Ureaplasma spp. and *M. hominis* recovered from urogenital specimens in a tertiary-care setting. Secondary objectives were to compare susceptibility patterns according to the organism detected and to explore unadjusted differences according to patient sex. Given the retrospective design and the absence of detailed clinical characterization for all patients, this study was designed as a microbiological surveillance analysis rather than an evaluation of treatment efficacy or antimicrobial resistance specifically among clinically confirmed genital infections.

## 2. Materials and Methods

### 2.1. Study Design, Patient Selection, and Specimen Collection

This retrospective, descriptive study was conducted in the Department of Microbiology at Medical Point Hospital. Clinical, demographic, and microbiological data were retrospectively retrieved from the hospital’s electronic information management system.

Between April 2023 and April 2025, a total of 242 patients underwent testing for genital Ureaplasma spp. and *Mycoplasma hominis* using the Mycoplasma IES kit (Autobio Diagnostics Co., Ltd., Zhengzhou, China). Patients were eligible for the final analysis when a urine or urethral swab specimen yielded a single-organism-positive result for Ureaplasma spp. or *M. hominis*, demonstrated sufficient microbial growth for antimicrobial susceptibility testing, and had valid antimicrobial susceptibility results available. Specimens demonstrating insufficient microbial growth and specimens showing mixed growth of Ureaplasma spp. and *M. hominis* were excluded. Following application of these criteria, 176 patients with single-organism-positive specimens were included in the final analysis.

Urine specimens were collected directly into sterile urine collection containers. Urethral specimens were obtained by physicians using sterile swabs and subsequently placed in transport medium. All specimens were transported to the microbiology laboratory within 1 h of collection and processed immediately upon arrival.

The study protocol was reviewed and approved by the Institutional Non-Interventional Clinical Research Ethics Committee of İzmir University of Economics (approval no. E-97429853-050.04-103174; committee decision/approval date: 11 July 2025). The official approval document was issued on 29 July 2025. The study was conducted in accordance with the Declaration of Helsinki. The requirement for informed consent was waived because of the retrospective nature of the study and the use of anonymized data. The corresponding official ethics document was issued on 29 July 2025.

### 2.2. Microbiological Identification and Antimicrobial Susceptibility Testing

Microbiological testing was performed using the Mycoplasma IES kit (Autobio Diagnostics Co., Ltd., Zhengzhou, China), a commercial culture-based system for the detection, semiquantitative enumeration, and phenotypic antimicrobial susceptibility testing of genital mycoplasmas.

Specimens were inoculated into the kit culture medium, and the resulting suspension was distributed into the corresponding wells of the 30-well test strip. The wells were overlaid with mineral oil to prevent evaporation, and the inoculated strips were incubated at 36–38 °C for 48 h before interpretation.

The assay detects microbial growth through biochemical reactions involving urea hydrolysis by Ureaplasma spp. and arginine metabolism by *M. hominis*. These reactions generate ammonia, increase the pH of the medium, and produce a visible color change from yellow to red. The 30-well strip contains dedicated wells for organism identification, semiquantitative enumeration, growth control, and antimicrobial susceptibility testing. Only specimens demonstrating sufficient growth according to the kit-defined semiquantitative threshold of ≥10^4^ CCU/mL were eligible for antimicrobial susceptibility analysis.

Antimicrobial susceptibility was assessed for 11 agents: clarithromycin, erythromycin, josamycin, roxithromycin, ciprofloxacin, ofloxacin, levofloxacin, tetracycline, minocycline, pristinamycin, and clindamycin.

The commercial panel did not include doxycycline or azithromycin; therefore, no susceptibility inference for these agents was made from results obtained with other tetracyclines or macrolides.

According to the Mycoplasma IES panel configuration, the tested concentrations were pristinamycin 2 mg/L; minocycline 2 and 8 mg/L; josamycin 2 and 8 mg/L; erythromycin 8 and 16 mg/L; roxithromycin 1 and 4 mg/L; clindamycin 0.25 and 0.5 mg/L; ofloxacin 1 and 4 mg/L; ciprofloxacin 1 and 2 mg/L; and clarithromycin 1 and 4 mg/L. Organism-specific concentrations were used for tetracycline (1 and 2 mg/L for Ureaplasma spp.; 4 and 8 mg/L for *M. hominis*) and levofloxacin (2 and 4 mg/L for Ureaplasma spp.; 1 and 2 mg/L for *M. hominis*).

For agents tested at two concentrations, absence of growth in both antimicrobial-containing wells was categorized as susceptible, growth at the lower concentration but not at the higher concentration was categorized as intermediate, and growth at both concentrations was categorized as resistant. For pristinamycin, which was tested at a single concentration of 2 mg/L, absence or presence of growth was categorized as susceptible or resistant, respectively. These classifications represent categorical phenotypic results generated according to the commercial assay’s interpretive algorithm and should not be interpreted as independently determined reference broth-microdilution MIC values. These assay-derived susceptibility percentages should not be considered directly interchangeable with resistance estimates derived from CLSI- or EUCAST-based reference MIC methods.

The culture-based Mycoplasma IES method does not reliably differentiate Ureaplasma urealyticum from Ureaplasma parvum. Although positive Ureaplasma results had been designated as U. urealyticum in the original laboratory records according to the kit reporting terminology, all such detections are reported as *Ureaplasma* spp. in the present study to accurately reflect the taxonomic resolution of the assay.

### 2.3. Statistical Analysis

Statistical analyses were performed using IBM SPSS Statistics for Windows, version 26.0 (IBM Corp., Armonk, NY, USA). Continuous variables were assessed for normality using the Shapiro–Wilk test and are presented as mean ± standard deviation (SD) or median (minimum–maximum), as appropriate. Categorical variables are presented as numbers and percentages.

Continuous variables with an approximately normal distribution were compared using the independent-samples *t* test. Categorical variables were compared using Pearson’s chi-square test or Fisher’s exact test, as appropriate according to expected cell frequencies. For categorical variables with more than two levels, overall comparisons were performed first; where applicable, post hoc pairwise comparisons of proportions were conducted using Bonferroni-adjusted Z tests.

Antimicrobial susceptibility categories (susceptible, intermediate, and resistant) were compared according to organism and patient sex. Because the distribution of organisms differed substantially according to sex, particularly because *M. hominis* was detected almost exclusively among women, sex-based analyses were considered exploratory and unadjusted and were not interpreted as demonstrating an independent effect of sex.

All statistical tests were two-sided, and *p* < 0.05 was considered statistically significant.

## 3. Results

### 3.1. Study Population and Microbiological Characteristics

During the study period, 242 patients underwent testing for Ureaplasma spp. and *M. hominis*. After exclusion of specimens with insufficient growth and mixed-organism-positive specimens, 176 patients with single-organism-positive specimens and valid antimicrobial susceptibility results were included in the final analysis.

The mean age of the included patients was 34.5 ± 7.5 years (range, 20–58 years). Of the 176 patients, 146 (83.0%) were women and 30 (17.0%) were men. Among the 146 women, 10 (6.8%) were pregnant at the time of specimen collection. A total of 125 (71.0%) specimens were genital/urethral swabs and 51 (29.0%) were urine specimens.

Ureaplasma spp. were detected in 145 patients (82.4%), whereas *M. hominis* was detected in 31 patients (17.6%). Because mixed cultures were excluded, these categories were mutually exclusive (Table 1 and Table 2).

### 3.2. Overall Antimicrobial Susceptibility Patterns

Marked differences were observed across the antimicrobial agents tested. The highest resistance rate was observed for clindamycin, with 175 of 176 isolates (99.4%) categorized as resistant by the commercial assay. Resistance was also high for ciprofloxacin (161/176, 91.5%) and ofloxacin (113/176, 64.2%).

In contrast, high phenotypic susceptibility was observed for josamycin (175/176, 99.4%), minocycline (174/176, 98.9%), pristinamycin (174/176, 98.9%), and tetracycline (148/176, 84.1%). Clarithromycin and erythromycin each showed an overall susceptibility rate of 73.9%, whereas levofloxacin susceptibility was 52.8%.

These findings represent categorical phenotypic susceptibility results obtained using the Mycoplasma IES system rather than reference MIC measurements.

### 3.3. Comparison According to Organism

The distribution of patient sex differed significantly according to organism (*p* = 0.032). *M. hominis* was detected in 30 women (96.8%) and one man (3.2%), whereas Ureaplasma spp. were detected in 116 women (80.0%) and 29 men (20.0%). No statistically significant differences between organism groups were observed for age, pregnancy status, or specimen type.

Marked organism-specific differences were identified for several antimicrobial agents. Clarithromycin susceptibility was 89.7% among Ureaplasma spp., whereas 30 of 31 *M. hominis*-positive results (96.8%) were resistant (*p* < 0.001). Similarly, 89.7% of Ureaplasma spp. were susceptible to erythromycin, whereas all 31 *M. hominis*-positive results were resistant (*p* < 0.001).

Roxithromycin demonstrated a similar pattern: 89.7% of Ureaplasma spp. were susceptible, whereas all 31 *M. hominis*-positive results were categorized as resistant (*p* < 0.001). Levofloxacin susceptibility was also significantly higher among Ureaplasma spp. than among *M. hominis* (62.1% vs. 9.7%, *p* < 0.001).

Clindamycin resistance was observed in all 145 Ureaplasma-positive results and in 30 of 31 (96.8%) *M. hominis*-positive results. No statistically significant between-organism differences were identified for ciprofloxacin, clindamycin, josamycin, minocycline, ofloxacin, pristinamycin, or tetracycline.

For the smaller *M. hominis* group (n = 31), Wilson 95% confidence intervals for selected resistance proportions were as follows: clarithromycin 96.8% (95% CI, 83.8–99.4%), erythromycin 100% (95% CI, 89.0–100%), levofloxacin 71.0% (95% CI, 53.4–83.9%), and clindamycin 96.8% (95% CI, 83.8–99.4%).

### 3.4. Exploratory Comparison According to Patient Sex

In the unadjusted sex-based analysis, women demonstrated higher resistance rates than men for clarithromycin (28.1% vs. 6.7%, *p* = 0.020), erythromycin (28.8% vs. 6.7%, *p* = 0.020), and roxithromycin (30.1% vs. 6.7%, *p* = 0.006). No statistically significant sex-related differences were identified for the other evaluated antimicrobial agents.

However, because 30 of the 31 *M. hominis*-positive results occurred in women and *M. hominis* demonstrated substantially greater macrolide resistance than Ureaplasma spp., these unadjusted findings should not be interpreted as demonstrating an independent association between female sex and antimicrobial resistance. This analysis was therefore considered exploratory. The exploratory sex-based susceptibility results are presented in Table 3.

## 4. Discussion

The present study provides contemporary local antimicrobial susceptibility data for Ureaplasma spp. and *M. hominis* recovered from urogenital specimens in a tertiary-care setting. The principal findings were very high phenotypic resistance rates to ciprofloxacin and ofloxacin, comparatively preserved activity of josamycin, minocycline, pristinamycin, and tetracycline, and marked organism-specific differences in susceptibility to several antimicrobial agents. These findings illustrate the heterogeneity of antimicrobial susceptibility among genital mycoplasmas and reinforce the importance of interpreting surveillance data according to both the organism detected and the antimicrobial agent evaluated [8,9,10,11,12,15].

Antimicrobial resistance among genital mycoplasmas varies substantially across geographic regions, study populations, periods of surveillance, and susceptibility-testing methodologies. A recent global systematic review and meta-analysis demonstrated considerable geographic heterogeneity in antimicrobial resistance among clinical Mycoplasma and Ureaplasma isolates, reinforcing the importance of contemporary regional surveillance rather than extrapolating susceptibility patterns from geographically distant populations [12]. Differences between studies may additionally reflect antimicrobial exposure, prescribing practices, patient characteristics, specimen type, organism distribution, and laboratory methodology [8,9,10,11]. Accordingly, the present results should primarily be interpreted as local phenotypic surveillance data rather than universally generalizable resistance estimates.

One of the most prominent findings was the high frequency of phenotypic fluoroquinolone resistance, particularly to ciprofloxacin (91.5%) and ofloxacin (64.2%). Reduced fluoroquinolone susceptibility among urogenital Mycoplasma and Ureaplasma isolates has been reported across multiple geographic regions [8,9,11,14,15]. Fluoroquinolone resistance is generally associated with alterations within the quinolone resistance-determining regions of DNA gyrase and topoisomerase IV, involving genes such as gyrA, gyrB, parC, and parE [12,14,15]. The high phenotypic resistance observed in our population may reflect local antimicrobial selective pressure; however, because molecular resistance determinants were not investigated, the underlying mechanisms cannot be established from the present data. Future studies combining phenotypic susceptibility testing with molecular characterization would help determine whether these phenotypes correspond to established resistance-associated mutations.

In contrast, high phenotypic susceptibility was observed for josamycin, minocycline, pristinamycin, and tetracycline. Previous surveillance studies have similarly demonstrated relatively preserved activity of several tetracycline-class agents, josamycin, and pristinamycin, although considerable geographic variability exists [8,9,11,16]. Systematic analyses of tetracycline resistance among *M. hominis* and Ureaplasma spp. have demonstrated substantial between-study heterogeneity, indicating that susceptibility to this antimicrobial class cannot be assumed to be uniform across populations [16]. Thus, the high susceptibility observed in our cohort provides useful local surveillance information but should not be interpreted as establishing clinical treatment efficacy. Treatment outcomes were not evaluated in this study, and in vitro susceptibility does not necessarily translate directly into clinical effectiveness.

Marked organism-specific differences were particularly evident for macrolide agents. *M. hominis* demonstrated substantially greater resistance to erythromycin, clarithromycin, and roxithromycin than Ureaplasma spp. This pattern is biologically plausible because *M. hominis* possesses well-recognized intrinsic resistance to 14- and 15-membered macrolides, whereas susceptibility differs for other macrolides and related agents [8,9,10,11,12,13]. Consequently, aggregate susceptibility estimates combining *M. hominis* and Ureaplasma spp. may obscure microbiologically important organism-specific differences.

A particularly notable finding was the very high rate of phenotypic clindamycin resistance generated by the commercial assay. Overall, 175 of 176 isolates were categorized as resistant, including all 145 Ureaplasma-positive results and 30 of 31 *M. hominis*-positive results. High clindamycin resistance among Ureaplasma isolates has been reported previously. Notably, a Turkish study using the same Mycoplasma IES (Autobio Diagnostics) system categorized all 82 evaluated Ureaplasma isolates as resistant to clindamycin [18]. The study used clindamycin concentrations of ≤0.25 and ≥0.5 mg/L, corresponding to the concentrations incorporated into the commercial system. This independent observation using the same assay provides external support for the very high clindamycin resistance detected among Ureaplasma spp. in our cohort.

The similarly high resistance observed among *M. hominis*-positive results requires greater caution. Published susceptibility patterns for *M. hominis* and clindamycin have not been uniformly consistent with the 96.8% resistance observed in our cohort. Differences in study population, isolate characteristics, antimicrobial concentrations, interpretive criteria, and susceptibility-testing methodology may contribute to discordant findings. The Mycoplasma IES system evaluates clindamycin at predefined concentrations and generates categorical phenotypic results according to the manufacturer’s interpretation algorithm. Because reference broth-microdilution MIC testing was not performed, the unexpectedly high resistance among *M. hominis*-positive results could not be independently confirmed. Therefore, while the Ureaplasma finding is supported by previous results obtained using the same commercial system, the 30/31 *M. hominis* result should be regarded as an assay-derived resistance signal rather than a confirmed resistance-prevalence estimate based on a reference MIC method, and it requires confirmation using standardized reference susceptibility testing [17].

Methodological differences are particularly important when comparing antimicrobial susceptibility studies of genital mycoplasmas. CLSI M43 describes standardized broth microdilution and agar dilution approaches for susceptibility testing of human mycoplasmas and Ureaplasma spp., including quality-control reference strains and MIC interpretive criteria for selected antimicrobial agents [17]. Commercial culture-based assays offer practical advantages in routine clinical laboratories by combining organism detection, semiquantitative estimation, and susceptibility categorization; however, categorical results generated by such systems are not necessarily interchangeable with independently determined reference MICs. Consequently, direct numerical comparisons between studies using different commercial systems or reference susceptibility methods should be made cautiously. The present results represent categorical phenotypic susceptibility results generated by the Mycoplasma IES system and should be interpreted within that methodological framework.

Another important consideration is the distinction between colonization and clinically significant infection. Ureaplasma spp. and *M. hominis* may be detected in the urogenital tract of individuals without attributable disease, and asymptomatic carriage is well documented [3,4,7]. The European STI Guidelines Editorial Board has emphasized that detection alone does not establish causality and has cautioned against indiscriminate testing and treatment of asymptomatic individuals [7].

In the present retrospective study, detailed information regarding presenting symptoms, specific clinical diagnoses, previous antimicrobial exposure, bacterial vaginosis, and concomitant sexually transmitted pathogens was not consistently available. Accordingly, our findings should not be interpreted as resistance patterns exclusively among patients with symptomatic genital infection. Rather, they describe the in vitro phenotypic susceptibility patterns of Ureaplasma spp. and *M. hominis* recovered from urogenital specimens submitted for testing at our institution. This distinction is particularly relevant to antimicrobial stewardship because unnecessary treatment of colonization may contribute to antimicrobial selective pressure without established clinical benefit [7].

The apparent sex-associated differences observed in the unadjusted analysis should also be interpreted cautiously. Women demonstrated higher resistance rates to clarithromycin, erythromycin, and roxithromycin; however, *M. hominis* was detected almost exclusively among women in our cohort. Because *M. hominis* itself demonstrated substantially greater resistance to these macrolide agents, the observed association between female sex and resistance is likely to be strongly influenced by the unequal distribution of organisms between women and men. Previous studies have also demonstrated sex-related differences in the prevalence and antimicrobial resistance distribution of *M. hominis* [19]. Therefore, the present findings do not establish female sex as an independent predictor of antimicrobial resistance. Larger datasets with adequate representation of both organisms across sexes and multivariable analyses would be required to determine whether sex independently contributes to susceptibility patterns.

A further methodological limitation concerns taxonomic resolution. The culture-based Mycoplasma IES system used in this study does not reliably differentiate Ureaplasma urealyticum from Ureaplasma parvum. Consequently, although positive Ureaplasma results had historically been recorded as U. urealyticum according to laboratory reporting terminology, they are reported as Ureaplasma spp. throughout the revised manuscript. This distinction is relevant because U. urealyticum and U. parvum may differ in prevalence and clinical associations [4,7]. Future investigations incorporating species-specific molecular identification would permit more precise characterization of local epidemiology and susceptibility patterns.

Several additional limitations should be considered. First, this was a retrospective, single-center study, which limits generalizability to other institutions and geographic regions. Second, susceptibility was determined using a commercial phenotypic system rather than standardized reference MIC testing, and molecular resistance mechanisms were not investigated. Third, specimens demonstrating insufficient growth and mixed Ureaplasma spp./*M. hominis* growth were excluded; therefore, the findings apply to single-organism-positive specimens meeting the predefined semiquantitative threshold. Fourth, detailed clinical variables, including symptoms, clinical diagnosis, previous antimicrobial exposure, concomitant infections, and subsequent treatment response, were not consistently available. Thus, associations between in vitro susceptibility and clinical outcomes could not be assessed. Fifth, the relatively small number of *M. hominis*-positive results (n = 31), particularly the presence of only one male patient with *M. hominis*, limits organism- and sex-stratified inference. Finally, because U. urealyticum and U. parvum could not be differentiated, species-specific resistance estimates within the Ureaplasma group could not be determined.

Despite these limitations, the study has several strengths. It provides contemporary local susceptibility data across a relatively broad panel of 11 antimicrobial agents and includes organism-specific comparisons between Ureaplasma spp. and *M. hominis*. The revised methodology provides substantially greater detail regarding specimen handling, culture conditions, semiquantitative inclusion criteria, antimicrobial concentrations, and the interpretation framework of the commercial assay. The study also identifies an unusually high local clindamycin-resistance signal that warrants further surveillance and highlights the importance of validating unexpected commercial-assay findings using standardized reference methods.

Future research should combine standardized reference antimicrobial susceptibility testing with species-specific molecular identification and characterization of resistance determinants [12,14,17]. Prospective multicenter studies incorporating clinical presentation, indication for testing, previous antimicrobial exposure, coinfections, bacterial vaginosis status, treatment selection, microbiological eradication, and clinical outcomes would allow a clearer distinction between colonization and clinically relevant infection [7]. Molecular or genomic investigation of established resistance-associated loci may further clarify the mechanisms underlying regional resistance patterns [12,14]. Finally, longitudinal surveillance using consistent testing methodologies would enable meaningful assessment of temporal trends and could support antimicrobial stewardship by identifying changes in local susceptibility profiles [8,11,12].

Taken together, the present findings should primarily be viewed as local in vitro phenotypic surveillance data. They demonstrate substantial organism- and antimicrobial-specific variation in susceptibility while also illustrating the methodological and clinical complexities inherent in interpreting genital mycoplasma testing. Local surveillance may complement, but should not replace, appropriate clinical assessment, careful distinction between colonization and infection, and standardized susceptibility testing when clinically indicated.

## 5. Conclusions

This retrospective single-center study provides local in vitro phenotypic surveillance data for Ureaplasma spp. and *Mycoplasma hominis* recovered from urogenital specimens. Resistance was particularly high for clindamycin, ciprofloxacin, and ofloxacin, whereas josamycin, minocycline, pristinamycin, and tetracycline showed comparatively preserved in vitro activity. Marked organism-specific differences were observed for several macrolides and levofloxacin. These findings should be interpreted as assay-derived surveillance results rather than evidence of clinical treatment efficacy. The inability of the commercial system to distinguish U. urealyticum from U. parvum, the lack of reference MIC confirmation, incomplete clinical characterization, and the exploratory nature of sex-based analyses limit causal and therapeutic inference. Future prospective studies combining species-specific molecular identification, standardized reference susceptibility testing, and clinical outcome data are warranted.

## Figures and Tables

**Table 1:** Demographic and specimen characteristics of the study population.

Characteristic	n/Mean ± SD	%/Median (Min–Max)
Age, years	34.53 ± 7.54	33 (20–58)
Sex		
Female	146	83.0
Male	30	17.0
Pregnancy among women (n = 146)		
No	136	93.2
Yes	10	6.8
Specimen type		
Genital/urethral swab	125	71.0
Urine	51	29.0
Organism		
Ureaplasma spp.	145	82.4
*Mycoplasma hominis*	31	17.6

**Table 2:** Overall phenotypic antimicrobial susceptibility results.

Antimicrobial	Susceptible, n (%)	Intermediate, n (%)	Resistant, n (%)
Ciprofloxacin	8 (4.5)	7 (4.0)	161 (91.5)
Clarithromycin	130 (73.9)	3 (1.7)	43 (24.4)
Clindamycin	1 (0.6)	0 (0)	175 (99.4)
Erythromycin	130 (73.9)	2 (1.1)	44 (25.0)
Josamycin	175 (99.4)	1 (0.6)	0 (0)
Levofloxacin	93 (52.8)	17 (9.7)	66 (37.5)
Minocycline	174 (98.9)	2 (1.1)	0 (0)
Ofloxacin	13 (7.4)	50 (28.4)	113 (64.2)
Pristinamycin	174 (98.9)	—	2 (1.1)
Roxithromycin	130 (73.9)	0 (0)	46 (26.1)
Tetracycline	148 (84.1)	8 (4.5)	20 (11.4)

**Table 3:** Exploratory distribution of phenotypic antimicrobial susceptibility according to patient sex.

Antimicrobial/Category	Female (n = 146)	Male (n = 30)	Test Statistic	*p* Value
Ciprofloxacin				
Susceptible	7 (4.8)	1 (3.3)	3.258	0.177
Resistant	135 (92.5)	26 (86.7)	3.258	0.177
Intermediate	4 (2.7)	3 (10)	3.258	0.177
Clarithromycin				
Susceptible	103 (70.5) ^a^	27 (90) ^b^	7.616	0.020
Resistant	41 (28.1) ^a^	2 (6.7) ^b^	7.616	0.020
Intermediate	2 (1.4) ^a^	1 (3.3) ^a^	7.616	0.020
Erythromycin				
Susceptible	102 (69.9) ^a^	28 (93.3) ^b^	7.498	0.020
Resistant	42 (28.8) ^a^	2 (6.7) ^b^	7.498	0.020
Intermediate	2 (1.4) ^a^	0 (0) ^a^	7.498	0.020
Josamycin				
Susceptible	145 (99.3)	30 (100)	-	1.000
Intermediate	1 (0.7)	0 (0)	-	1.000
Levofloxacin				
Susceptible	72 (49.3)	21 (70)	4.191	0.135
Resistant	58 (39.7)	8 (26.7)	4.191	0.135
Intermediate	16 (11)	1 (3.3)	4.191	0.135
Minocycline				
Susceptible	144 (98.6)	30 (100)	-	1.000
Intermediate	2 (1.4)	0 (0)	-	1.000
Ofloxacin				
Susceptible	9 (6.2)	4 (13.3)	1.988	0.411
Resistant	95 (65.1)	18 (60)	1.988	0.411
Intermediate	42 (28.8)	8 (26.7)	1.988	0.411
Pristinamycin				
Susceptible	144 (98.6)	30 (100)	-	1.000
Resistant	2 (1.4)	0 (0)	-	1.000
Roxithromycin				
Susceptible	102 (69.9)	28 (93.3)	-	0.006
Resistant	44 (30.1)	2 (6.7)	-	0.006
Tetracycline				
Susceptible	122 (83.6)	26 (86.7)	0.102	1.000
Resistant	17 (11.6)	3 (10)	0.102	1.000
Intermediate	7 (4.8)	1 (3.3)	0.102	1.000

Note: Sex-based analyses are exploratory and unadjusted because organism distribution differed substantially by sex. Clindamycin is reported in the overall and organism-specific analyses; given near-universal resistance (175/176), it was not emphasized as a sex-comparison endpoint. Different superscript letters indicate significant pairwise differences after Bonferroni adjustment where applicable.

## Data Availability

The data that support the findings of this study are available from the corresponding author upon reasonable request. The data are not publicly available due to patient privacy and ethical restrictions.
